# Oral health problems in high-performance athletes at 2019 Pan American Games in Lima: a descriptive study

**DOI:** 10.1038/s41405-021-00078-1

**Published:** 2021-06-16

**Authors:** Catalina Opazo-García, Jeel Moya-Salazar, Karina Chicoma-Flores, Hans Contreras-Pulache

**Affiliations:** 1 Asociación de Odontología Deportiva Chile, Santiago, Chile; 2 School of Medicine, Faculties of Health Science, Universidad Norbert Wiener, Lima, Peru; 3 Centro de Documentación e Investigación “Pedro Ortiz Cabanillas”, Lima, Peru; 4 Hospital Nacional Docente Madre Niño San Bartolomé, Lima, Peru

**Keywords:** Calculus, Gum disease

## Abstract

**Introduction:**

Dental care is provided for high-performance athletes at national and international sports events. Elite athletes may seek care for sports-related injuries and pre-existing oral diseases. Previous studies indicate an association between oral health problems and negative performance impacts in elite athletes.

**Objectives:**

To determine the prevalence of the most common oral pathologies in high-performance athletes during the emergency dental care performed at the Lima 2019 Pan American Games (JPL-19).

**Methodology:**

All reports of athletes (≥18 years old, of both sexes, from 41 countries) who received emergency dental care at Pan American Villas during the JPL-19 were included. Injuries and types of oral diseases were classified according to the Injury and Disease Surveillance System proposed by the International Olympic Committee.

**Results:**

Of the 6680 participating athletes, 76 (1.14%) presented as dental emergencies, 90.8% (69/76) of the athletes seen presented pre-existing oral pathological conditions, the most frequent were periodontal diseases (34%, 26/76) and dental caries (29%, 22/76). Among the sports with the most cases, there were 22 (29%) in athletics, 6 (8%) in soccer, and 6 (8%) in taekwondo. The most frequent dental emergencies came from Peru, Puerto Rico, Bahamas, Grenada, and Venezuela.

**Conclusions:**

Pre-existing oral diseases were more frequent than sports-related accidents. The most prevalent diseases were periodontal disease and dental caries disease. It is necessary to implement new care strategies for athletes, based on prevention, before and during sports competitions.

## Introduction

Sports Dentistry has traditionally been related to the use of mouthguards,^1^ although the relevance of this discipline is to promote prevention that would lead to an integral treatment of patients (highly competitive athletes) so they can represent their countries in national and international sports events without being affected by any kind of oral health problem.^2^ In this regard, oral pathologies are associated with poor athletic performance, as recently have been suggested.^3^

Oral health problems in athletes can affect their performance, for example, malocclusion can cause posture problems, pain in the jaw, neck, back, as well as breathing problems impairing rest and muscle repair during sleep.^4,5^ On the other hand, periodontal diseases can directly affect muscle recovery and consequently sports performance, especially in high-performance athletes with a weakened immune system associated with high levels of cortisol due to being under permanent stress and demand,^3,6^ presenting pain from deep cavities; for example, it has been associated with an 18% decrease in sports performance that can lead to partial or total disability to compete.^2^ Furthermore, if left untreated it can trigger a disseminated infection beyond the damaged tooth, causing an accumulation of pus and affecting the well-being of athletes.^2,3^

More than 50 years ago, at the 1958 FIFA World Cup in Sweden, Mario Trigo, pioneer of Sports Dentistry, reported a high frequency of pre-existing oral diseases due to lack of prevention in athletes. He performed 118 extractions in players from different countries, to prevent a complicated infection.^4^ At the Athens 2004 Olympic Games, after physiotherapy, the second most requested service by athletes in the medical area was dental care.^7^ On the other hand, at the London 2012 Olympic Games, it was found that the oral health of Olympic athletes was poor due to substantial prevalence of dental caries with 55%, and periodontal diseases where 76% presented gingivitis and 14% periodontitis.^2^ Before Rio 2016, Dutch elite athletes’ oral health was examined. The study concluded that almost 50% of them needed dental regular treatment and proposed that oral health screening incorporated into the general preventive health care of elite athletes is necessary to ensure athletes are fully healthy during competitions like the Olympic and Paralympic Games.^8^ In the year 2018 in the United Kingdom was studied the first representative sample of athletes from different sports areas to evaluate the oral condition and the performance impact. More than three-quarters (77%) of athletes had gingival bleeding and 49% presented established caries. This study concluded that oral diseases and associated negative performance impacts are prevalent in British elite and professional athletes. Regular screening and the use of effective oral health promotion strategies may minimize performance impacts from poor oral health.^9^

Studies on Sports Dentistry have not yet been carried out in Latin America, especially during the Pan American Games. We aimed to describe oral health problems at the Lima 2019 Pan American Games (JPL-19), determining the number of orofacial injuries and oral diseases in elite athletes who participated in this sports event and demonstrate that the oral problems are mainly caused by previous oral diseases and not by orofacial trauma.

## Material and methods

### Study design

A secondary data review, with a descriptive cross-sectional design, was performed during JPL-19 (26 July – 11 August 2019).

### Eligibility criteria and setting

Athletes who received dental care services at the implemented surgery in the sports villages were included. The inclusion criteria were ≥18 age, of both sexes, from all participating delegations (41 countries and 6680 high-performance athletes) who required emergency dental care (dental care services).^10^ The athlete assessment period began 4 days before the start of the event and ended 1 day later (22 July – 12 August 2019). The data declared in medical records were digital. A dentist per sports village was provided; the time spent per patient for diagnosis and treatment was unlimited according to the patient’s need. Clinical and complementary examinations were performed (periapical radiography and in some cases medical radiography were used to evaluate the temporomandibular joint). Simultaneously with the care, all the data were registered digitally. The dentist in charge of providing the care to patients was responsible for the registration.

### Clinical evaluation

A digital data sheet was filled out per patient. Domains of information included athlete personal data, type of sport, plus X-rays, consultations, and prescriptions. The assessment of the patient began with an extraoral examination done by a qualified dentist. Then, the oral cavity in general was examined, and finally, the affected area was specifically evaluated. As an emergency clinical evaluation, the tools used were South Carolina periodontal probe in case of periodontal disease to determine if the patient had gingivitis or periodontitis (>3 mm depth on probing) and the presence of dental caries was determined by clinical and radiographic examination in deep caries without a specific measurement index.

### Data collection

The digital system of medical records, implemented ad hoc for the JPL-19, was adapted to the requirements of the Injury and Disease Surveillance System proposed by the International Olympic Committee (IDSS-IOC) that has been used since 2008.^11,12^ The IDSS-IOC is a standardized method specifically designed to register incidences of injury and illness in high-performance multi-sport events.^13^ The main limitation of the IDSS-IOC is that it is not specifically designed to monitor dental pathology, which means that limited data are available.

### Data management and analysis

All oral diseases were classified according to the International Code of Diseases (ICD-10). In addition, descriptive statistics and measures of central tendency were used. Data analysis was performed in IBM SPSS v22.0 (Armonk, US) for Windows.

## Results

**Fig. 1:**
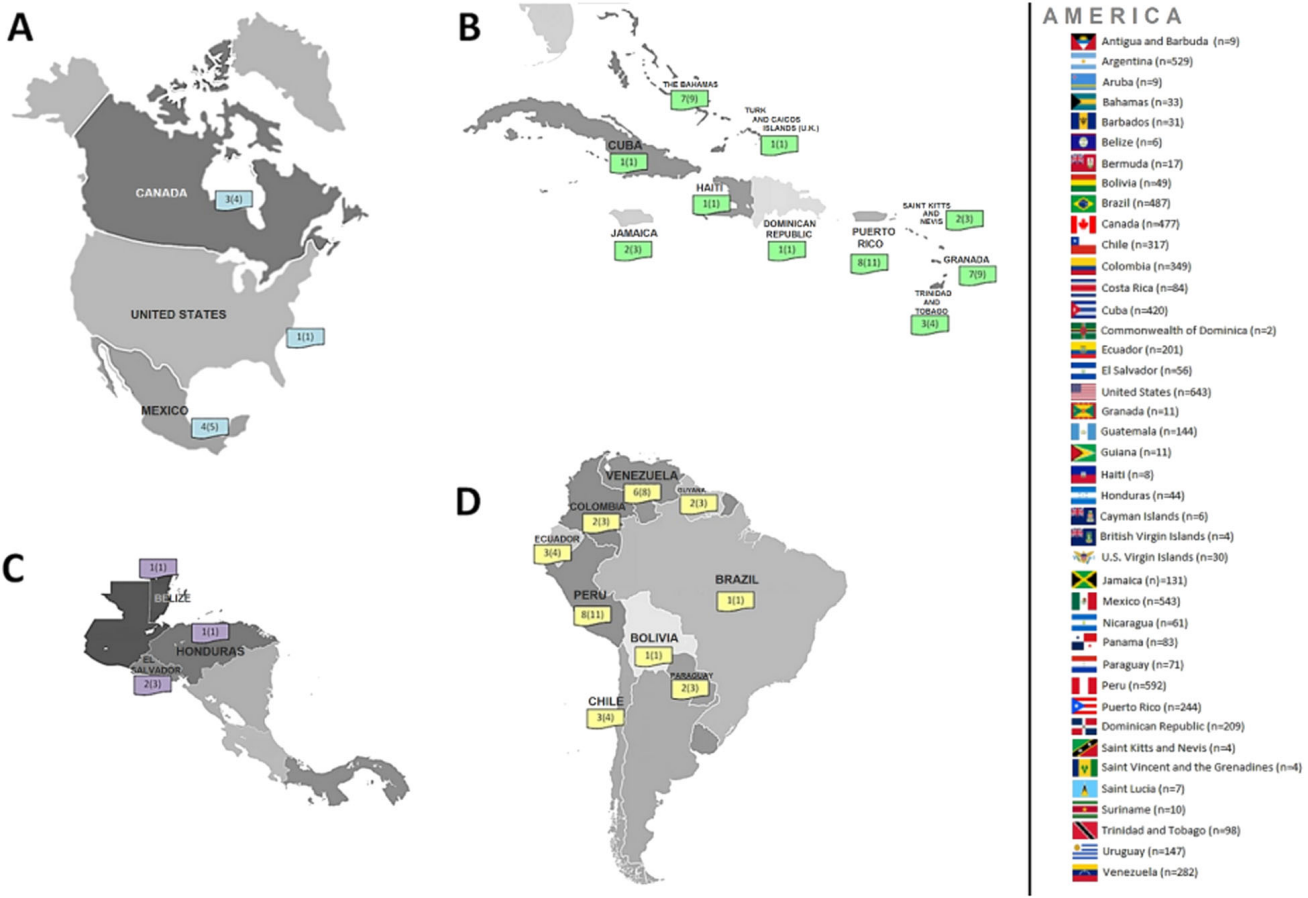
Demographic distribution of athletes with oral problems during the JPL-19. The map shows the number of cases according to different geographical regions including North (**A**) and South America (**D**), and Central America (**B**) and Caribbean (**C**). The percentages of total reported cases are in brackets. The “*n*” in the legend represents the total of athletes per country.

In the JPL-19, a total of 6680 athletes from 41 countries participated. Seventy-six (1.14%) athletes attended the dental emergency room (Fig. 1). These athletes were from 26 countries, mainly from the Caribbean (*n* = 33, 43.4%) and South America (*n* = 28, 36.8%).

The athletes went to the dental emergency service of their village to receive oral care and solve the current problem in their oral cavity. Countries with the most significant number of athletes who received emergency dental care were (Fig. 1): Peru 11% (8/599 athletes), Puerto Rico 11% (8/249); Bahamas (7/33) and Grenada (7/11) both 9%, and Venezuela 8% (6/282). The countries with the highest prevalence of dental emergencies were Grenada 64% and Bahamas 21%.

Of the 39 sports included in JPL-19, 25 sports (64%) reported athletes seen in the dental emergency service and 14 sports (36% of the total) did not report athletes with problems in the oral cavity. Of the 25 sports, 76 athletes went to the dental emergency services of their respective sports village. The highest number of athletes attended in the dental emergency service was 22 (29%) from athletics, and 6 (8%) from both soccer and taekwondo (Table 1).

**Table 1:** Clinical diagnosis of oral diseases during the JPL-19 according to athlete category.

Sport	Clinical diagnosis																				
	K007	K011	K02	K021	K036	K040	K041	K046	K05	K050	K051	K053	K055	K056	K061	K116	R520	S024	S025	Z464	Total (%)
Athletics				11	1				1	2	5				1	1					22 (29)
Handball												1							2		3 (4)
Baseball				1							2										3 (4)
Bowling						1															1 (1)
Mountain Biking						1															1 (1)
Road cycling																	1				1 (1)
Bodybuilding														2							2 (3)
Soccer				1			1				2		1							1	6 (8)
Hockey									1									1			2 (3)
Judo				1																	1 (1)
Karate				1																	1 (1)
Wrestling				2															1		3 (4)
Basque pelota			1						1												2 (3)
Racquetball											1										1 (1)
Rowing									1												1 (1)
Softball						1								1							2 (3)
Squash																			1		1 (1)
Surf			1											3							4 (5)
Taekwondo	1			2				1								2					6 (8)
Tennis				2																	2 (3)
Triathlon				1									1								2 (3)
Sailing	1												1								2 (3)
Volleyball															1						1 (1)
Beach Volleyball								1						1							2 (3)
Others		1					1				1		1								4 (5)
Total (%)	2 (3)	1 (1)	2 (3)	22 (29)	1 (1)	3 (4)	2 (3)	2 (3)	4 (5)	2 (3)	11 (14)	1 (1)	4 (5)	7 (9)	2 (3)	3 (4)	1 (1)	1 (1)	4 (5)	1 (1)	76 (100)

K007: tooth eruption; K011: impacted teeth; K02: dental caries; K021: dental caries; K036: dental calculus; K040: pulpitis; K041: pulp necrosis; K046: periapical abscess with fistula; K05: gingivitis and periodontal diseases; K050: acute gingivitis; K051: chronic gingivitis; K053: chronic periodontitis; K055: other periodontal diseases; K056: periodontitis; K061: gingival hyperplasia; K116: mucocele; R520: acute pain; S024: fracture of the malar and maxillary bone fracture; S025: tooth fractures; Z464: test and adjustment of orthodontic appliances.

Oral diseases are presented considering the ICD-10 Data in *N* (%).

There were various clinical conditions considered as dental emergencies. Athletes came from their country of origin with these pre-existing diseases, the most frequent were periodontal diseases (34%) and dental caries (29%). Various periodontal diseases were grouped into periodontal affections (ICD: K05, K050, K051, K53, K055, 056) independently of their severity and chronicity showed in Table 1. Dental caries disease was grouped into two clinical entities (K02 and K021).

Ninety percent of the athletes seen in the emergency room presented the following clinical dental conditions: dental caries disease (29%), periodontal diseases (34%), salivary gland mucocele (4%), pulpitis (4%), chronic periapical abscess (3%), among others (Table 1). On the other hand, only 9.2% presented a new condition, acute pain, tooth fracture, fracture of the malar and maxillary bone fracture, test and adjustment of orthodontic appliances.

## Discussion

This is the first study describing the oral health of athletes in the JPL-19, the main clinical conditions presenting as dental emergencies were associated with periodontal diseases and dental caries disease. In 9/10 athletes who received treatment, the conditions were pre-existing and 1/10 presented injuries during the sports event, such as trauma associated with the maxillofacial area. Of 41 participating countries, more than half of athletes from these countries presented dental emergencies, while of 39 sports, almost two-thirds of athletes from these countries expressed pain or discomfort during the sports event. The most affected disciplines were athletics (29%), soccer (8%), and taekwondo (8%); according to their origin Peru (11%), Puerto Rico (11%), Bahamas (9%), Granada (9%), and Venezuela (8%).

As Sports Dentistry is an emerging field in Latin America, this study represents the first effort to describe the problems presented in the JPL-19. In this respect, this contribution is added to the list of studies conducted in Latin America.^4,5,14–19^ Furthermore, this study was based on the official data of the sports event, and it had as a reference source the monitoring system of the Olympic Committee (SVLE-IOC) that provides standardized information for sports events and has been widely used in previous studies. Despite the information system was conceived by using a medical-clinical perspective, dental variables in the data delivered at the JPL-19 were not considered because few data about oral health were available. Thus, the aim of performing these examinations during JPL-19 was only for emergency purposes. This is one of the reasons for the low number of dental examinations or surgeries performed (*n* = 76).

It would be expected that in a sports event the dental emergencies are associated with oral trauma. On the contrary, this study shows that 90.8% of the emergency services were due to pre-existing diseases and only 9.2% were due to traumatic accidents or others. Our findings show that 1.14% of athletes received emergency dental care, which could hide the seriousness of the problem because the acute expression of an oral disease is generally late. The majority of the most prevalent pathologies are asymptomatic and of slow development, this could be explained by the public health infrastructure of each country, since very few countries in Latin America are able to get their high-performance athletes to go for regular check-ups with dentists.

In 2004, the Olympics in Athens identified oral health as an important public health problem.^7^ As previously evidenced in the 2012 London Olympics estimated that more than 50% of athletes had dental caries lesions (from incipient to profound) and more than 70% presented periodontal disease. A standardized care protocol was determined for all athletes and a card designed to diagnose the oral condition of the competitors was proposed.^2^ However, this tool has not been replicated in the following sports events; this is also the case for JPL-19.

In 2017, it was proposed to the Olympic Committee of each registered country in the Tokyo Olympic Games^15^ to develop an exhaustive review of 6 months before the sports event to treat periodontal diseases, caries disease, endodontic pathologies, pericoronitis as well as the fabrication of mouthguards. This way, the risk factors to achieve a good performance in the 2020 Olympic Games (now changed to 2021 due to the COVID-19 pandemic) in high-performance athletes would be controlled. Also, in 2021, Junior Pan American Games will be held in Cali, Colombia and in 2023 the Pan American and Para-pan American Games will be held as well in Santiago de Chile. According to this, we propose to carry out a 1-year control before the sports event to organize all the pertinent interventions and achieve a correct sports performance, without problems before and during the event, which would reduce the cost of not having an athlete in competition with his full potential.

Supporting the previous literature, oral health of elite athletes is poor. Prevalence of trauma is much lower than prevalence of common dental diseases. It highlights the need for regular oral health screening outsides of competition to detect dental diseases at an early stage and provide treatment and/or preventive care and advice.^8,9,20^

High-performance athletes with a weakened immune system due to demanding physical activity are more susceptible to widespread diseases; therefore, it is highly important to keep athletes healthy so that they do not have issues during training and competition, since muscle repair could be affected by an oral infection and disable the athlete from participating in a sports event.^16,21^

## Conclusion

Of all athletes attending, 1.14% were seen in the dental clinic. Of this 1.14% (76 athletes), 90.8% attended for pre-existing conditions (most commonly periodontal disease and dental caries). The countries with the highest number of cases were the Bahamas and Grenada. In addition, athletics, soccer, and taekwondo had a significant number of athletes with dental emergencies and pre-existing diseases.

In upcoming sports events, an information and data registration system that takes into account the requirements of Sports Dentistry should be used, plus a management protocol as in London 2012; and dismiss (or at least begin to discuss) the necessity of a restrictive access rule as used in JPL-19.
